# Protective Immunization of Atlantic Salmon (S*almo salar* L.) against Salmon Lice (*Lepeophtheirus salmonis*) Infestation

**DOI:** 10.3390/vaccines10010016

**Published:** 2021-12-23

**Authors:** Haitham Tartor, Marius Karlsen, Rasmus Skern-Mauritzen, Adérito Luis Monjane, Charles McLean Press, Christer Wiik-Nielsen, Rolf Hetlelid Olsen, Lisa Marie Leknes, Karine Yttredal, Bjørn Erik Brudeseth, Søren Grove

**Affiliations:** 1 Norwegian Veterinary Institute, 1433 Ås, Norway; haitham.tartor@vetinst.no (H.T.); aderito-luis.monjane@vetinst.no (A.L.M.); 2 PHARMAQ AS, P.O. Box 267 Skøyen, 0213 Oslo, Norway; marius.karlsen@zoetis.com (M.K.); christer.wiik-nielsen@zoetis.com (C.W.-N.); Rolf.Hetlelid-Olsen@zoetis.com (R.H.O.); lisa-marie.leknes@zoetis.com (L.M.L.); Karine.Yttredal@zoetis.com (K.Y.); 3 Institute of Marine Research, 5005 Bergen, Norway; rasmus.skern@hi.no; 4 Department of Preclinical Sciences and Pathology, Norwegian University of Life Sciences, 1430 Ås, Norway; charles.press@nmbu.no

**Keywords:** salmon lice, *Lepeophtheirus salmonis*, Atlantic salmon, vaccination, lice-gut proteins, P33

## Abstract

Vaccination against salmon lice (*Lepeophtheirus salmonis*) is a means of control that averts the negative effects of chemical approaches. Here, we studied the immunogenicity and protective effect of a vaccine formulation (based on a salmon lice-gut recombinant protein [P33]) against *Lepeophtheirus salmonis* infestation in Atlantic salmon in a laboratory-based trial. Our findings revealed that P33 vaccine can provide a measure of protection against immature and adult salmon lice infestation. This protection seemed to be vaccine dose-dependent, where higher doses resulted in lower parasitic infestation rates. We also provide immunological evidence confirming that P33-specific immune response can be triggered in Atlantic salmon after P33 vaccination, and that production of P33-specific antibodies in blood can be detected in vaccinated fish. The negative correlation between P33-specific IgM in salmon plasma and salmon lice numbers on vaccinated fish suggests that protection against lice can be mediated by the specific antibody in salmon plasma. The success of P33 vaccination in protecting salmon against lice confirms the possibility of employing the hematophagous nature of the parasite to deliver salmon-specific antibodies against lice-gut proteins.

## 1. Introduction

Salmon lice (*L. salmonis)* are crustacean ectoparasites that infest both farmed and wild salmonids stocks [1]. The feeding activity of the parasite causes skin damage and fin erosion [2], leaving salmon farms with massive economic losses resulting from managerial and therapeutic costs [3]. Salmon lice infestations are currently the most important biological limitation to salmon farming [4], and the continued expansion of salmon aquaculture will require adequate and sustainable control measures against the parasite [5]. Inspired by successful vaccination efforts against ectoparasites in mammals and the production of the first commercial vaccine against tropical tick (*Boophilus microplus*) in cattle (Gavac; Heber Biotec) [6], vaccination of salmon against salmon lice has been proposed as an alternative approach to controlling salmon lice infestations [7]. Under field conditions, the Gavac tick vaccine has proved the effectiveness of using a single tick antigen (Bm86) for the control of cattle tick infestations. The vaccine-induced protection from Gavac was achieved through the direct effect on tick fertility and was correlated with Bm86-specific antibody titers in blood [8]. The specific function of Bm86 within ticks has, however, not been completely elucidated. According to Rand et al. [9], Bm86 could have several epidermal growth factor-like domains and their dysfunction by specific antibodies against BM86 might interfere with the regulation of cell growth in ticks.

Developing effective vaccines against parasites is generally very challenging, as exemplified by several high-profile parasites including malaria [10], sheep blowfly [11], and sheep scab [12]. In the case of ectoparasites, the relative physical separation between pathogen and host makes it even more challenging. Because the ectoparasite is predominantly outside of the host, it is able to conceal a considerable portion of its constituents (i.e., potential antigens) from the host immune system, thus placing these “out of reach” of host defense mechanisms [13]. Allowing these concealed antigens to be “visible” to the host immune system could, in principle, lead to specific protective immune response against them. This possibility was successfully demonstrated by the Gavac vaccine, where the tick gut antigen (Bm86), which is concealed from the cattle’s immune system, proved to be an effective vaccine component.

Depending on the life stage, salmon lice can feed on skin mucus, epithelium, and/or blood [14]. This feeding pattern can directly expose concealed antigens in the lice gut to therapeutically induced immune components in the salmon blood and mucus. Given this possibility, intestinal lice antigens may therefore constitute potentially important targets for a vaccine strategy [15,16]. The search for such lice antigens has been the aim of a number of studies reported over the last decade [17,18,19,20,21]. In 2015, the first salmon lice vaccine against *Caligus rogercresseyi* lice was launched in Chile. This vaccine was based on a synthetic peptide and was reported to reduce the parasite load by 73% in vaccinated fish [22]. In 2020, another vaccine targeting salmon lice ribosomal protein (P0) showed a relative percentage of protection of 21% against the adult stage of *L. salmonis* [23]. Targeting P0 protein in salmon lice resulted in a significant impact on gravid female lice count with consequences on their reproductive efficacy, as evidenced by delayed hatching, and low copepodid counts in the F1 generation. In a previous study [24], we evaluated the protective effect of several salmon lice gut antigens including delta-like protein (P21), bifunctional heparin sulfate N-deacetylase/N-sulfotransferase (P37), putative Toll-like receptor 6 (P30), and potassium chloride, amino acid transporter (P33). The latter showed the most promising protective effect.

Here we report results from detailed testing of the immune mechanisms underlying the observed protection induced by P33. We hypothesize that P33 protection against salmon lice can be mediated by P33-specific antibodies in fish blood either by directly impairing P33 function in situ in the salmon lice gut or by initiating a complement-mediated lice gut cell damage.

## 2. Material and Methods

### 2.1. Experimental Facility and Ethics

This experiment (FOTS ID: 8733) was conducted at Industrial and Aquatic Laboratory (ILAB; Bergen, Norway) in 2017. Fish were handled according to the Norwegian regulations for use of fish as laboratory animals.

### 2.2. Vaccine Preparation

The lice P33 antigen (Ag) was produced as a HIS-tagged recombinant protein in *Escherichia coli* (SINTEF AS). P33 vaccines were formulated as water-in-oil emulsions containing the P33 antigen in 2 concentrations (0.1 mg/mL and 0.5 mg/mL). All the details of the recombinant P33 production and vaccine formulation have been previously described by Contreras et al. [24]. The *M. viscosa* control vaccine contained formalin-inactivated *Moritella viscosa* and was formulated similarly as the P33 vaccine.

### 2.3. Fish and Design of the Trial

**Figure 1:**
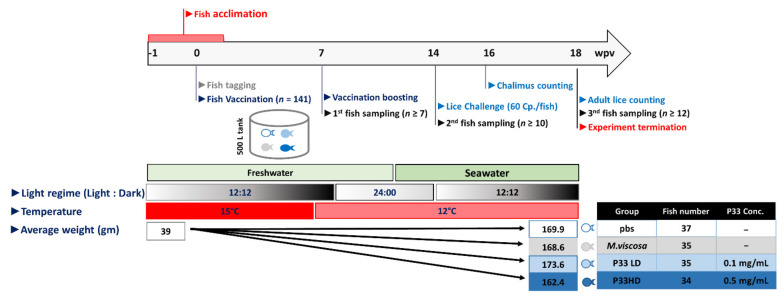
A schematic diagram showing the experimental design and the timeline of the P33 vaccination trial. Salmon fish were acclimatized for one week before they were tagged and vaccinated with P33 vaccine (low and high doses; LD and HD, respectively) or injected with pbs and *M. viscosa* vaccine (as a negative controls) at 1 wpa (0 wpv). At 8 wpa (7 wpv), the fish in the LD and HD were given a booster dose of P33 vaccine, and fish in the negative control groups were injected with the same material used in the first injection. All fish were challenged with lice copepodid at 14 wpv, and the chalimus and adult stages of sea lice were counted at 16- and 18-wpv, respectively. wpa: weeks post-acclimatization, wpv: weeks post-vaccination, Cp: copepodid. Conc.: Concentration, PBS: phosphate-buffered saline, LD: low dose, HD: high dose.

The experimental design of the trial is shown in Figure 1. In short, Atlantic salmon (*Salmo salar*) fish (*n* = 141; average weight of 39 g) were acclimatized for 1 week in circular flow-through tanks (volume = 500 L). Using a common garden design, fish were divided into 2 P33-vaccinated groups and 2 negative control groups (with PBS injection as non-immunized control and *Moritella viscosa* injection as a non-specific immunization control). Fish were tagged with a combination of maxillae and/or adipose fin clipping and PIT-tags (12 mm i-Tag 162 PIT, UID, Helsingborg, Sweden). All vaccination and boosting procedures were performed while the fish were in freshwater. The P33 groups were vaccinated twice, at 1- and 8-weeks post-acclimatization (wpa). At each vaccination, fish received a single intraperitoneal (IP) injection (100 µL) of vaccine containing P33 at either 0.1 mg/mL (low dose [LD]; *n* = 35) or 0.5 mg/mL (high dose [HD]; *n* = 34). For the two negative control groups, fish were intraperitoneally injected twice, i.e., at 1 and 8 wpa, with 100 µL of either phosphate-buffered saline (PBS; *n* = 37) or a monovalent vaccine against *Moritella viscosa* (*n* = 35). Even though the experiment has another replicate (another tank with similar four groups), only one tank was used for the purpose of this study. Fish were kept in freshwater (<0.5 ‰ salinity) for the first 10 weeks of the experiment. For the following eight weeks, the salinity was gradually raised to 34 ‰ over the remaining experimental period. During the first 5 weeks of the experiment, the water temperature was kept at 15 °C, and for the rest of the experiment, the fish were gradually adapted to 12 °C. Fish were fed a common commercial diet (Skretting AS, Stavanger, Norway) using an automatic feeder, and fish feeding was stopped 1 day before procedures involving vaccination, challenge, or sampling. Tricain Pharmaq (PHARMAQ AS, Oslo, Norway) was used to anesthetize (100 mg/L water) or euthanize (200 mg/L.) fish.

### 2.4. Lice Challenge Procedures

Fish in the four experimental groups (*n* ≥ 12/group) were bath-challenged once in a common garden setup at 14 weeks post-vaccination (wpv) using salmon lice copepodids (LsGulen; ILAB strain). During the procedure, the water depth of the tank was reduced to approximately 15 cm, and copepodites (60 copepodids/fish) were added to the tank and left with fish for 45 min before the water flow and volume were returned to normal levels. Throughout the challenge experiment, the salinity was maintained at 34 ‰, and the water temperature at 12 °C.

### 2.5. Lice Counts

Fish in the four experimental groups (*n* ≥ 12/group) were anesthetized when counting chalimus and euthanized when adult lice were counted. During counting, the whole surface of the fish was thoroughly examined, and a quick examination of the inside of the operculum and surface of gills aimed to locate the hidden parasites. The number of chalimus per fish was counted 2 weeks after the infestation (at 16 wpv). About 2 weeks later, when the chalimus had molted into the adult stage (at 18 wpv), the experiment was terminated, and the numbers of adult male and female lice per fish were counted. In anticipation of adult lice detaching themselves from fish in response to the anesthesia, fish were euthanized individually in separate buckets and both the numbers of detached and attached lice were counted. The number of lice and their developmental stage were compared with a negative control group kept in the same tank. The relative percentage protection (RPP) was calculated for each vaccinated group, using the formula RPP = 100 × (1 − [lice count in vaccinated group/lice count in the control group]).

### 2.6. Fish Sampling

Plasma and tissue samples (spleen, anterior kidney, and skin from around the lateral line below the dorsal fin) were collected from fish (*n* ≥ 10/group) in each of the four experimental groups at 7, 14, and 18 wpv. However, the tissue samples collected from 3 fish in the HD group at 7 wpv were lost before analysis. Blood samples were collected from the caudal vein using vacutainer tubes with heparin (VACUETTE^®^, Greiner Bio-One, Frickenhausen, Germany) and were put on ice until they were centrifuged (2000× *g*, 5 min, at room temperature) to prepare plasma samples. All tissues were excised and cut in half. One half was placed in RNAlater (Ambion, Inc., Austin, TX, USA) and kept at 4 °C for 24 h, and then stored at −20 °C until further use. The other half was fixed in 10% neutral buffered formalin for 24 h before transfer to 70% ethanol. Formalin-fixed tissues from the PBS and HD groups were then paraffin-embedded and cut into 3 μm sections. The analyses performed on the collected blood and tissue samples, as well as the numbers of samples in each analysis are summarized in Supplementary Table S1.

### 2.7. RNA Isolation and cDNA Synthesis

Total RNA was extracted from 30–40 mg of each tissue sample using RNeasy Mini Kit (Qiagen GmbH, Hilden, Germany) for anterior kidney and spleen tissues, and a combination of Trizol (Qiagen, Valencia, CA, USA) and RNeasy Mini Kit for skin tissues, following the manufacturers’ instructions. RNA was eluted in 40 µL of RNAse-free H_2_O and the concentration and purity of RNA were determined by spectrophotometry using NanoDrop ND2000 (Thermo Fisher Scientific Inc, Waltham, MA, USA). Samples were stored at −80 °C until further use. Contaminating DNA was removed, and 1 µg of total RNA was used for cDNA synthesis, by using the QuantiTect Reverse Transcription Kit (Qiagen, Hilden, Germany) according to the manufacturer’s instructions. Stock solutions were prepared by diluting the cDNA 5 times by adding 80 µL RNAse free H_2_O, and finally, cDNA was stored at −80 °C until further use.

### 2.8. RT-qPCR Protocol

Spleen, anterior kidney, and skin samples (*n* ≥ 7/group) from the HD and PBS control groups, collected at 7, 14 and 18 wpv, were analyzed by RT-qPCR for immunoglobulin genes (*sIgM*, *mIgM*, *IgT-C* [a third sub-isotype of IgT in Atlantic salmon, Accession number: GenBank HQ379938.1]*, IgD, sIgT-A*, *mIgT-A*, *sIgT-B,* and *mIgT-B*). Samples collected at 18 wpv were obtained from lice-infested fish after counting adult lice. A pilot experiment was performed to optimize the cDNA concentration in expression analysis of the different genes using samples from control fish (injected with PBS). Accordingly, anterior kidney and spleen cDNA were diluted 1:10 to analyze the expression of *sIgM*, *mIgM*, *IgT-C* and *IgD,* whereas a 1:3 dilution was used to analyze *sIgT-A*, *mIgT-A*, *sIgT-B* and *mIgT-B*. To analyze the expression of these exact genes in skin tissues, the cDNA samples were diluted 1:3 and 1:1 for the former and latter list of immunoglobulin genes, respectively. The RT-qPCR experiments were performed using the CFX384 instrument (Bio-Rad Laboratories GmbH, Germany) with SsoAdvanced™ Universal SYBR^®^ Green Supermix (Bio-Rad Laboratories, Hercules, CA, USA). Each sample was analyzed in triplicate, using a total reaction mix volume of 6 µL per well (2 µL cDNA, primers [sequences are shown in Table 1] at 244 nM and 3.7 µL of Green Supermix). Elongation factor 1α (*EF1α*) gene was solely used as a reference gene based on the work performed on previous studies [25,26]; however, the cDNA amount used to analyze *EF1α* expression was adjusted according to the gene and the tissue analysed. Non-template wells with H_2_O, primers, and SsoAdvanced™ Universal SYBR^®^ Green Supermix were run on each plate as a negative control. The following thermocycling conditions were used: initial denaturation (30 s at 95 °C) followed by 40 cycles of denaturation (15 s at 95 °C), annealing (30 s at 60 °C), and extension (5 s at 55 °C). Finally, a melting curve was made by measuring the fluorescence during a temperature range of 60–95 °C to confirm the specificity of the end-product amplicon in the reaction. Fluorescence was measured, expressed as relative fluorescence units (RFU) and quantification cycles (Cq) for every reaction that was measured. Real-time data were analyzed using the CFX Manager software version 3.1 (Bio-Rad Laboratories, Hercules, CA, USA). All samples with Ct values ≥ 40 were not considered for further gene expression analysis. The *EF1α* expression was confirmed to be stable before its values were used to normalize the immunoglobulin gene expression values [27], resulting in -∆Ct values (Ct target genes-Ct *EF1α*). Mann–Whitney U-test was used to analyze the differences in ∆Ct values between the PBS and P33-vaccinated groups at each time point (α = 0.05).

### 2.9. Enzyme-Linked Immunosorbent Assay (ELISA)

The level of P33-specific IgM antibodies in plasma was quantified using ELISA as described by Contreras et al. [24]. The plasma samples (*n* ≥ 10/group) collected from fish in the three experimental groups (pbs, P33 LD and P33 HD) at 7, 14 and 18 wpv were analyzed (at 1:100 dilution) against the recombinant P33 antigen (1 µg/mL) in duplicate. The 4C10 mAb (this Ab is raised against rainbow trout IgM and recognizes salmon IgM subtype A [IgM-A; [28]]) and F1-18 mAb (this Ab is raised against purifiedrainbow trout plasma IgM and recognizes salmon IgM subtypes A and B [29]) were used as secondary antibody (1:20) in parallel analyses in this study. Horseradish peroxidase-conjugated sheep anti-mouse IgG (ECL, NA931V; 1:2000) was used as tertiary antibody.

Color development was stopped by adding 50 µL of 2 M H_2_SO_4_ and absorbance was read at 450 nm using an ELISA plate reader (Multiscan EX, Artisan; Thermo Electron Corporation, Vantaa, Finland)).

### 2.10. Immunohistochemistry (IHC)

#### 2.10.1. Analysis of Total IgM and Production of IgM Positive Cells

The abundance of secreted IgM and IgM positive (IgM^+^) cells in spleen and anterior kidney tissues were compared between the HD and PBS groups at 14 wpv. For this, we performed IHC analysis of IgM using the EnVision^+^ kit (Dako), according to the manufacturer’s instructions. Briefly, tissue sections (*n* ≥ 8/group) were dewaxed, rehydrated, and epitopes were demasked using heat retrieval protocol in a citrate buffer (pH 6) [30]. Endogenous peroxidase was inhibited (3% H_2_O_2_ in methanol; 10 min) and sections were blocked using 5% BSA in Tris buffer saline (TBS). Slides were incubated with anti-salmon IgM mAb F1-18 (1:500 in Tris buffer [TB] with 1% BSA) overnight at 4 °C, and slides without the anti-IgM antibody were used as a negative control. HRP labeled anti-mouse antibody EnVision^TM^ system (Dako, K4005) was used as a secondary antibody for 30 min at room temperature. AEC^+^ High Sensitivity Substrate Chromogen Ready-to-Use (Dako; K3469) was used for 10 min for signal development and the reaction was stopped by washing the slides in running tap water. Counterstaining was performed using Mayer’s hematoxylin (Chemi Teknikk, 5B-535) for 15 s and slides were mounted using Aquatex^®^ (Merck, Poole, UK). The labeling intensity of secreted IgM and the abundance of IgM^+^ cells were analyzed based on the principles described by Deshmukh et al. [31], and each slide was given intensity and abundance scores according to a scoring system defined in Table 2. For negative controls, the above protocol was performed on spleen and anterior kidney sections from the pbs and P33 HD group, omitting only anti-IgM antibody or omitting only HRP labeled anti-mouse antibody.

#### 2.10.2. Analysis of P33-Specific Antibodies In Situ in Spleen

A modified IHC protocol was developed to demonstrate the presence of P33-specific antibodies *in situ* in spleen tissue sections. The protocol, which we named *inverted IHC* (In.IHC), applies HIS-tagged recombinant P33 protein as a “primary antibody” (or “bait” for P33-specific antibodies) and a rabbit anti-HIS antiserum as “secondary antibody”. Spleen tissue sections from fish (*n* ≥ 10/group) in the HD and PBS group at 14 wpv were treated exactly as in the standard IHC protocol described above, up to the point where the sections were blocked. After blocking, the slides were incubated overnight at 4 °C with HIS-tagged recombinant P33 protein (50 µL per section, 1µg/mL P33 in TB with 1% BSA). Sections were then washed thoroughly with TBS and then incubated with rabbit anti-HIS-tag polyclonal antibody (MicroMol GmbH, Germany) diluted 1:500 in TB with 1% BSA (1 h, room temperature). After the unbound antibody was washed off, the sections were incubated with HRP labeled anti-rabbit antibody EnVision^TM^ system (Dako, K4009) for 30 min at room temperature. As in the IHC above, staining was developed and stopped, counterstain applied, and the slides mounted. For negative controls, the above protocol was performed on spleen sections omitting only the bait, omitting only secondary antibody, or omitting only HRP labeled anti-rabbit antibody or with only HIS-tagged recombinant proteins (P12 and P30) [24].

## 3. Results

### 3.1. P33 Vaccination Reduces Salmon Lice Counts on Atlantic Salmon

**Figure 2:**
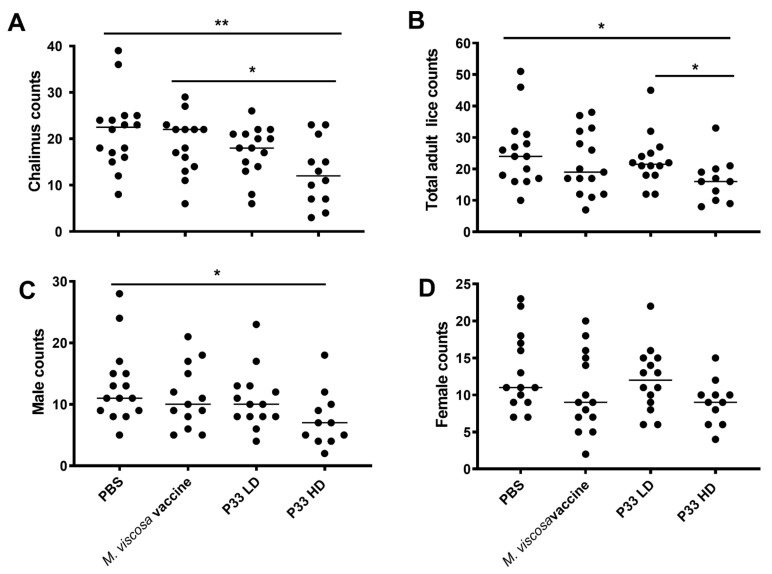
High dose P33 vaccination reduces lice numbers on vaccinated fish. Column scatter plots of lice count in mock-vaccinated (PBS [phosphate-buffered saline].and *M. viscosa* vaccine) and P33-vaccinated (LD [low dose], and HD [high dose]) Atlantic salmon fish after lice challenge. (**A**) Chalimus counts (16 weeks post-vaccination [wpv]), (**B**) Total adult lice counts (18 wpv), (**C**) Male counts and (**D**) Female counts. Horizontal lines in panels (**A**,**B**) represent the median lice counts in different groups. Asterisks denote significant differences (* *p* < 0.05; ** *p* < 0.01).

Chalimus and adult lice counts differed significantly between the experimental groups (Figure 2). Using a Kruskal–Wallis test followed by Dunn’s *post hoc* test (α = 0.05), and Bonferroni correction of *p* values, the HD group was shown to have a significantly lower chalimus count compared to both the PBS and *M. viscosa* injected groups (*p* = 0.004 and 0.02, respectively; Figure 2A). The number of adult lice was significantly lower in the HD group as compared to the PBS and LD groups (*p* = 0.02, for both; Figure 2B). The separated male and female counts (Figure 2C,D) revealed that the significant difference in the total adult count between the pbs and P33 HD groups was attributed to the male counts more than to the female counts. Compared with the PBS group, the number of chalimus lice on fish in the LD and HD groups was, respectively, reduced by 19.0 and 41.3%, whereas total adult lice counts were, respectively, reduced by 11.1 and 35.7%. Also, an RPP against adult females was estimated at 4.1 and 28.5%, respectively, in the LD and HD groups. Interestingly, the comparison of the LD and HD groups with the *M. viscosa* group showed reduced chalimus numbers by 14.1 and 41.2%, respectively. However, total adult lice count in LD and HD groups were lower than that in the *M. viscosa* group by—5.1 and 23%, respectively.

### 3.2. P33 Vaccination Induces P33-Specific IgM Antibodies

#### 3.2.1. Quantification of P33-Specific IgM in Plasma

**Figure 3:**
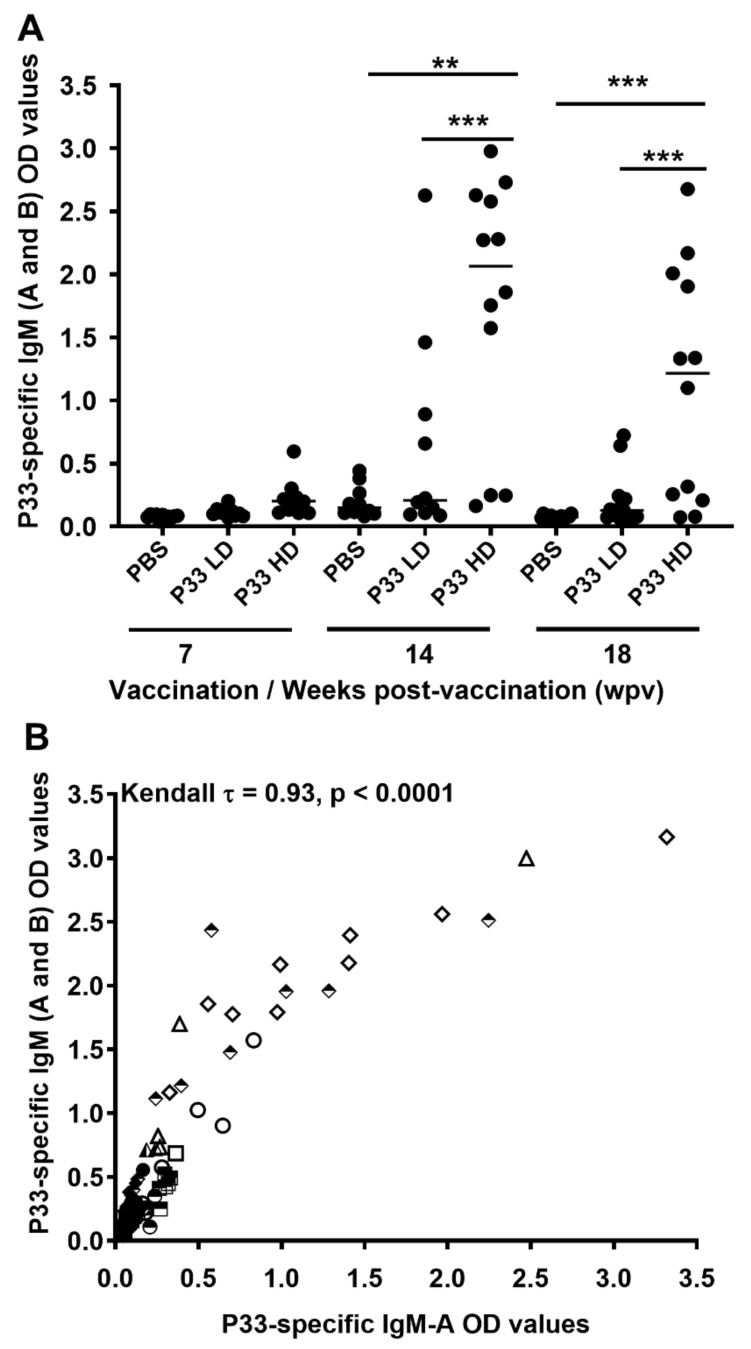
P33 vaccination induces specific antibody response in the plasma of vaccinated fish. (**A**) Column scatter plot of ELISA OD values representing P33-specific IgM Ab (A and B subtypes) in plasma of mock-vaccinated (injected with phosphate-buffered saline [PBS]) or P33-vaccinated (low-dose [LD] consisted of 0.1 mg/mL P33 orhigh-dose [HD] with 0.5 mg/mL P33) groups, at 7-, 14-, and 18-weeks post-vaccination (wpv). Horizontal lines on the plot represent the median OD values in different groups. Asterisks denote significant differences (** *p* < 0.01; *** *p* < 0.001). (**B**) Scatter plot of levels of P33-specific IgM subtype A antibody (OD values; *x*-axis) versus levels of P33-specific IgM antibody subtype A and B (OD values; *y*-axis) showing the correlation between P33-specific IgM-A and IgM (A and B subtypes) ELISA OD values in plasma of P33-vaccinated (LD and HD) and mock-vaccinated (PBS and *M. viscosa*) fish collected at 7 (solid symbols), 14 (open symbols) and 18 (half-open symbols) wpv.Kendall’s rank correlation coefficient (τ) and *p*-value are shown.

The IgM-A and -B ELISA analysis showed that there were no significant differences (Kruskal–Wallis test) between the experimental groups at 7 wpv (Figure 3A). At 14 wpv, Kruskal–Wallis test followed by Dunn’s *post hoc* showed that the HD group had significantly higher levels of P33-specific antibodies (OD; 450 nm) than both the LD (*p* = 0.006) and PBS (*p* = 0.002) groups, respectively (Figure 3A). A similar difference was observed at 18 wpv, with increased statistical significance for both differences (*p* < 0.0001; Figure 3A). Interestingly, fish in the group HD group had P33-specific IgM OD values average at 18 wpv lower than that measured at 14 WPV; yet, the difference seemed to be non-significant (*p* = 0.11; data not shown). The data at 14 and 18 wpv together suggest a dose-response effect of P33 vaccination in salmon. The IgM-A ELISA analysis of the plasma samples confirmed these differences (data not shown). A Kendall’s rank correlation analysis of OD values from the two ELISA analyses showed that the level of P33-specific IgM-A antibodies was strongly correlated with the level of P33-specific IgM-A and -B antibodies (Kendall τ = 0.93, *p* < 0.0001) (Figure 3B), suggesting that the IgM-B antibody response was not detectably different from the IgM-A response. All *p* values in the multiple comparisons were adjusted using Bonferroni test.

#### 3.2.2. Demonstration of P33-Specific Antibodies In Situ in the Spleen of Vaccinated Fish

**Figure 4:**
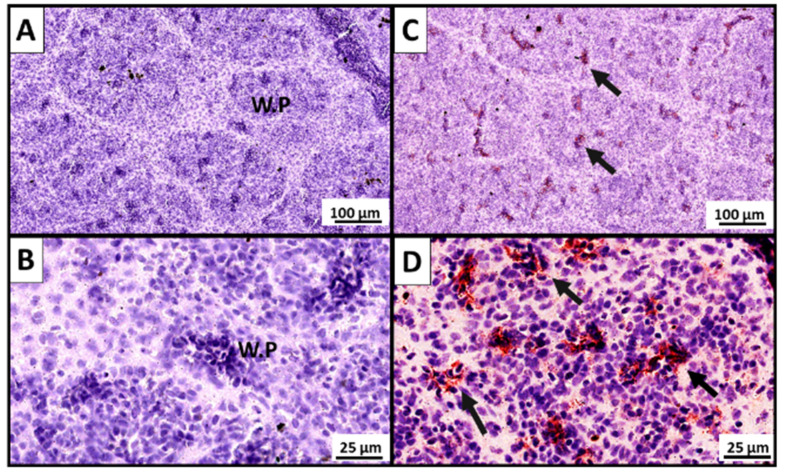
Detection of P33-specific antibodies in the spleen of P33-vaccinated fish. A modified immunohistochemistry protocol (inverted IHC; In.IHC) was used to demonstrate localization of P33-specific antibodies in spleen tissue of mock-vaccinated (injected with phosphate-buffered saline) fish (**A**,**B**; *n* ≥ 10) and fish injected with high dose of P33 (**C**,**D**; *n* ≥ 10) at 14 weeks post-vaccination. The pictures shown in panels (**A**,**C**) are from fish with plasma P33-specific IgM (A and B subtypes) OD values of 0.2 and 3.0, respectively. The red labeling spots (black arrows) show the binding of recombinant P33-protein to hypothesized P33-specific antibodies in the white pulp (WP) areas of spleen tissue of the P33-vaccinated fish in association with blood vessels.

At 14 wpv, P33-specific labeling was detected in the spleen of fish in the HD group using In.IHC (Figure 4C,D), but not in the PBS control group (Figure 4A,B), or the other negative control sections (Supplementary Figure S1). The P33-specific labeling was confined to the white pulp areas of the spleen and was most apparent in association with blood vessels (Figure 4C,D).

### 3.3. Immunoglobulin Kinetics Are Altered after P33 Vaccination

#### 3.3.1. P33 Vaccine Modifies Immunoglobulin Gene Expression in Vaccinated Fish

**Figure 5:**
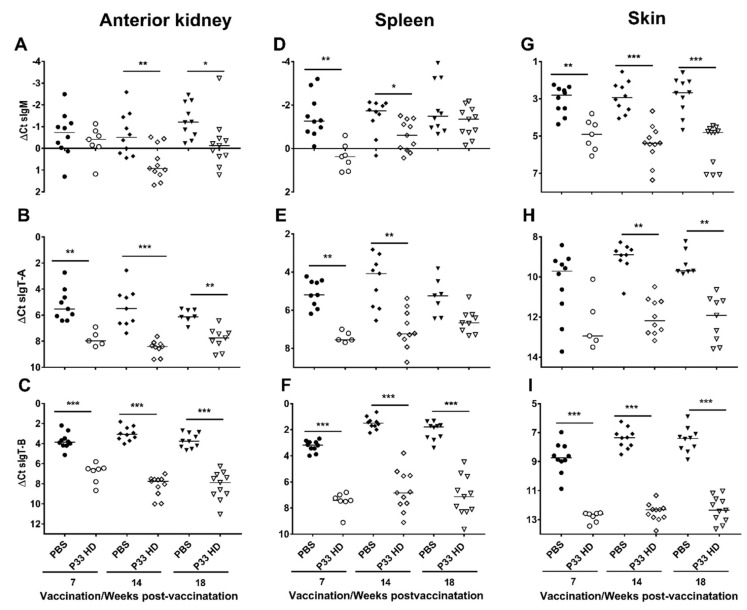
Transcript levels of secretory immunoglobulin genes decrease in the anterior kidney, spleen, and skin in response to P33 vaccination. Column scatter plots of normalized Ct (∆Ct) values from the RT-qPCR analysis representing the expression of *sIgM* (**A**,**D**,**G**), *sIgT-A* (**B**,**E**,**H**), and *sIgT-B* (**C**,**F**,**I**) in anterior kidney (**A**–**C**), spleen (**D**–**F**), and skin (**G**–**I**) of mock-vaccinated (injected with phosphate-buffered saline [PBS; filled symbols]) and P33-vaccinated (high dose [HD; open symbols]) groups at 7, 14 and 18 weeks post-vaccination. The mRNA levels were normalized against *EF1α* and each dot on the plot represents one fish. Asterisks denote significant differences (* *p* < 0.05; ** *p* < 0.01; *** *p* < 0.001), and horizontal lines on the plots show the median of ∆Ct values.

**Figure 6:**
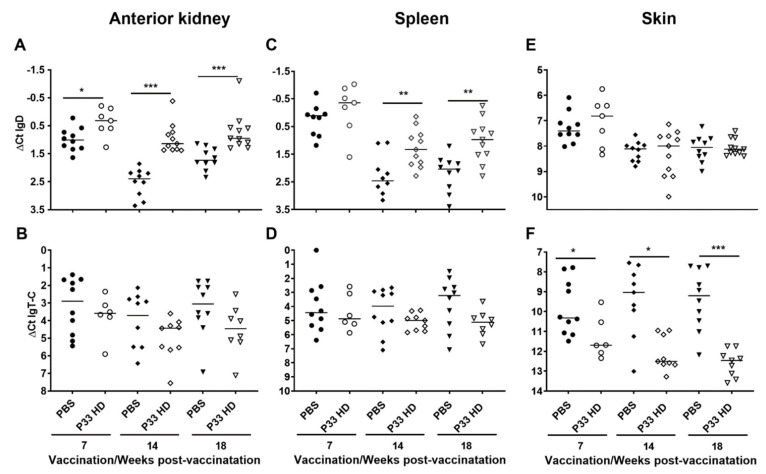
IgD transcript levels increase in the anterior kidney and spleen and IgT-C levels decrease in the skin in response to P33 vaccination. Column scatter plots of normalized Ct (∆Ct) values from the RT-qPCR analysis, representing the expression of *IgD and IgT-C* in anterior kidney (**A**,**B**), spleen (**C**,**D**), and skin (**E**,**F**) of mock-vaccinated (injected with phosphate-buffered saline [PBS; filled symbols]) and P33-vaccinated (high dose[HD; open symbols]) groups at 7-, 14- and 18-weeks post-vaccination. The mRNA levels were normalized against *EF1α* and each dot on the plot represents one sample from one fish. Asterisks denote significant differences (* *p* < 0.05; ** *p* < 0.01; *** *p* < 0.001) and horizontal lines on the plots show the median of ∆Ct values.

**Figure 7:**
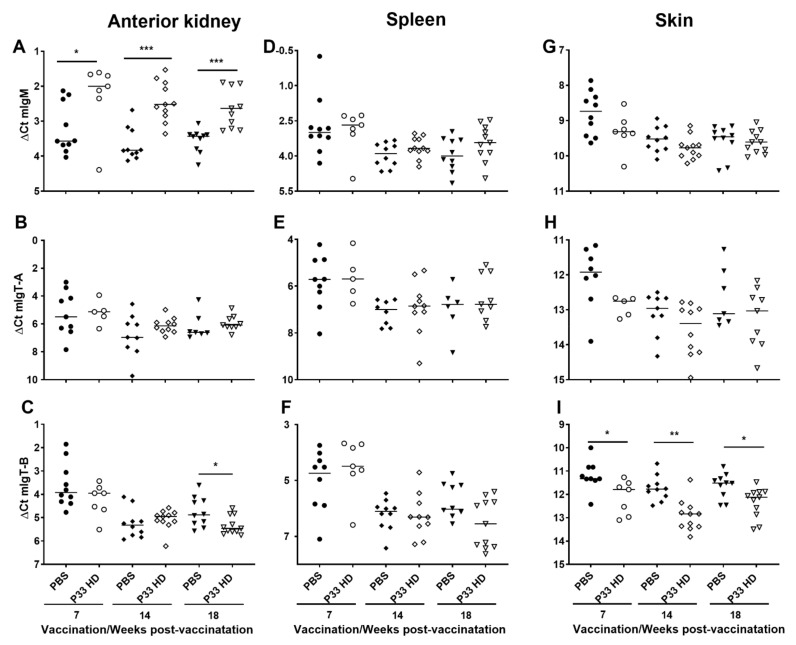
Increase in transcript levels of membrane-bound IgM (*mIgM*) in anterior kidney and decrease in transcript levels of membrane-bound IgT-B (*mIgT-B*) in the skin in response to P33 vaccination. Column scatter plots of normalized Ct (∆Ct) values from the RT-qPCR analysis, representing the expression of *mIgM* (**A**,**D**,**G**), *mIgT-A* (**B**,**E**,**H**) and *mIgT-B* (**C**,**F**,**I**) in anterior kidney (**A**–**C**) spleen (**D**–**F**), and skin (**G**–**I**) of mock-vaccinated (injected with phosphate-buffered saline [PBS; filled symbols]) and P33-vaccinated (high dose [HD; open symbols]) groups at 7, 14 and 18 weeks post-vaccination. The mRNA levels were normalized against *EF1α* and each dot on the plot represents one sample from one fish. Asterisks denote significant differences (* *p* < 0.05; ** *p* < 0.01; *** *p* < 0.001).

The secretory immunoglobulin (*sIg*) genes, including *sIgM*, *sIgT-A,* and *sIgT-B* and *IgT-C* (hypothesized to be secretory immunoglobulin based on its truncated heavy chain), were in all examined tissues and at all timepoints expressed at higher transcript levels in the PBS group (Figure 5 and Figure 6). For *sIgM* and *sIgT-A*, these differences were, with few exceptions, statistically significant. For *sIgT-B* all the differences were statistically significant (Figure 5). For *IgT-C*, the differences were statistically significant in the skin but not in the anterior kidney and spleen (Figure 6). In contrast, the transcript levels of the membrane-bound immunoglobulin (*mIg*; including *mIgM*, *mIgT-A*, *mIgT-B*) and *IgD* genes varied between the tissues (Figure 6 and Figure 7). For *mIgM*, the transcript levels of HD fish were significantly higher than for PBS fish in anterior kidney at all sampling points (Figure 7A). No such differences were seen in the spleen and skin (Figure 7D,G). For *mIgT-A*, no significant differences between the vaccine groups were observed (Figure 7B,E,H). Transcript levels of *mIgT-B* were significantly lower in HD fish compared with PBS control fish in the skin at all sampling times (Figure 7I) and in the anterior kidney at 18 wpv (Figure 7C), a difference not seen in the spleen (Figure 7F). For *IgD*, the transcript levels were generally higher in the HD group (Figure 6). This tendency was significant in the anterior kidney at all sampling times and in the spleen at 14 and 18 wpv (Figure 6A,C).

#### 3.3.2. IgM Production in Fish Immune Tissues Is Modulated after P33 Vaccination

**Figure 8:**
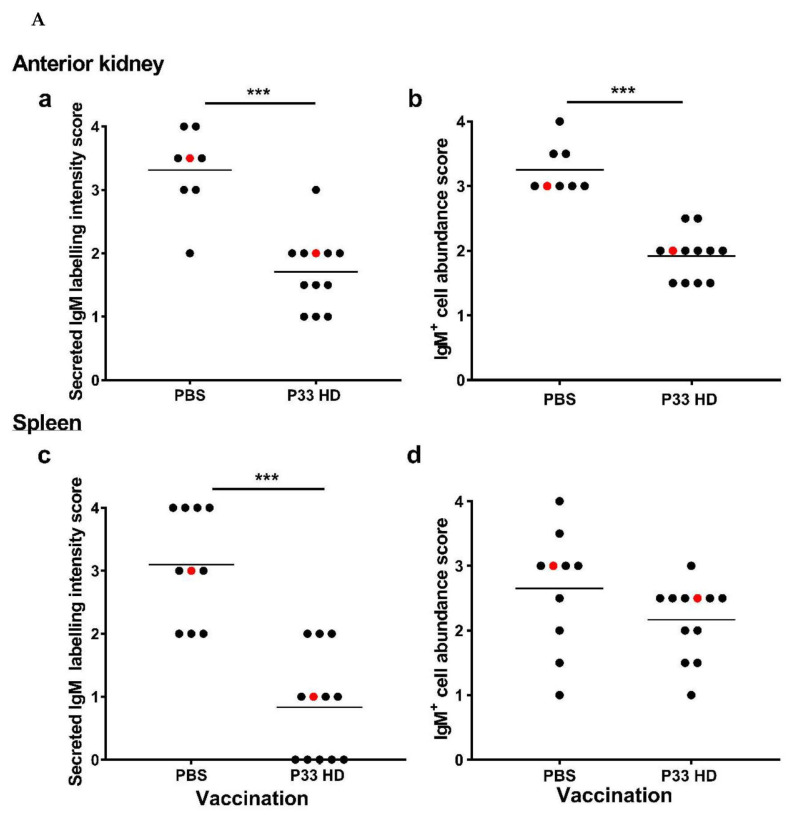

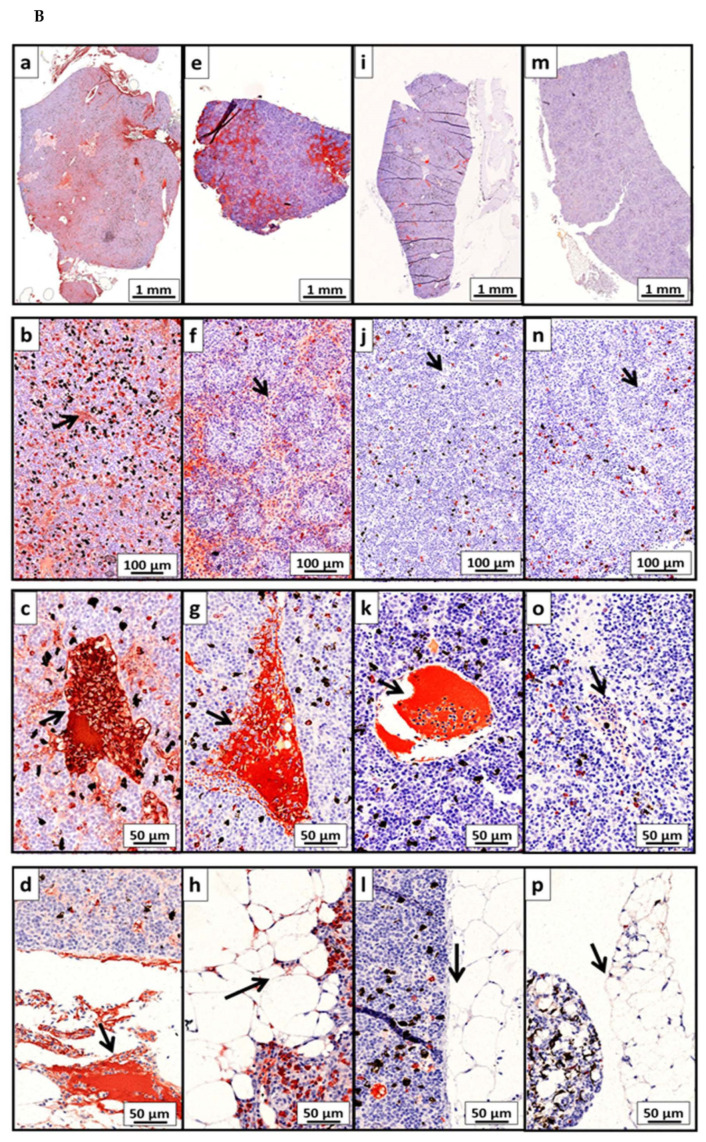
Immunohistochemical analysis of IgM P33-vaccinated fish. (**A**) Column scatter plots showing the difference in IHC scoring of secreted IgM labeling intensity (**a**,**c**) and abundance of IgM+ cell (**b**,**d**) in anterior kidney (*n* ≥ 8/group) and spleen (*n* ≥ 10/group) between the mock-vaccinated (injected with phosphate-buffered saline [PBS]) and P33-vaccinated (high dose [HD]) fish at 14 weeks post-vaccination (wpv). Each dot represents the average score of five microscopic fields on one tissue section obtained from one fish, and the red dots represent tissue sections in the pictures in panel ‘B’. Asterisks denote significant differences *** *p* < 0.001) and the horizontal lines on the plots show the median values. (**B**) IgM labeling (red color) in IHC demonstrating the difference in intensity and distribution of secreted and cell-associated IgM in the anterior kidney (**a**–**d**,**i**–**l**) and spleen (**e**–**h**,**m**–**p**) between PBS-injected (**a**–**h**) and P33-vaccinated (**i**–**p**) fish at 14 wpv. The images show IgM staining in the whole tissue section (**a**,**e**,**i**,**m**; 2× magnification), the splenic red pulp (arrows in **f**,**n**; 20× magnification), sinusoids in anterior kidney (arrows in **b**,**j**; 20× magnification), large blood vessels (arrows in **c**,**g**,**k**,**o**; 40× magnification), and the visceral tissue around spleen and kidney (arrows in **d**,**h**,**l**,**p**; 40× magnification).

The IHC results showed that the PBS group had a significantly higher median score for secreted IgM in both spleen and anterior kidney in comparison with the HD group (Mann-Whitney U-test, *p* = 0.0007 and 0.0002, respectively; Figure 8A(a,c). The secreted IgM labeling could be observed in association with the red pulp of the spleen, the sinusoids of the anterior kidney, blood in large blood vessels, and the adipose tissue of both spleen and anterior kidney (Figure 8B). The median score of IgM^+^ cell density in the spleen and anterior kidney was higher in PBS group fish as compared to HD group fish, though the difference was only significant in the anterior kidney (Mann-Whitney U-test, *p* = 0.0002; Figure 8A(b,d)). In both HD and PBS groups, IgM^+^ cells were generally scattered through the white pulp in spleen tissue and the parenchyma of the anterior kidney (Figure 8B). No IgM-specific labeling was detected on negative control sections (Supplementary Figure S2).

### 3.4. Vaccine-Induced Protection against Salmon Lice Correlates with the P33-Specific IgM in Fish Plasma

**Figure 9:**
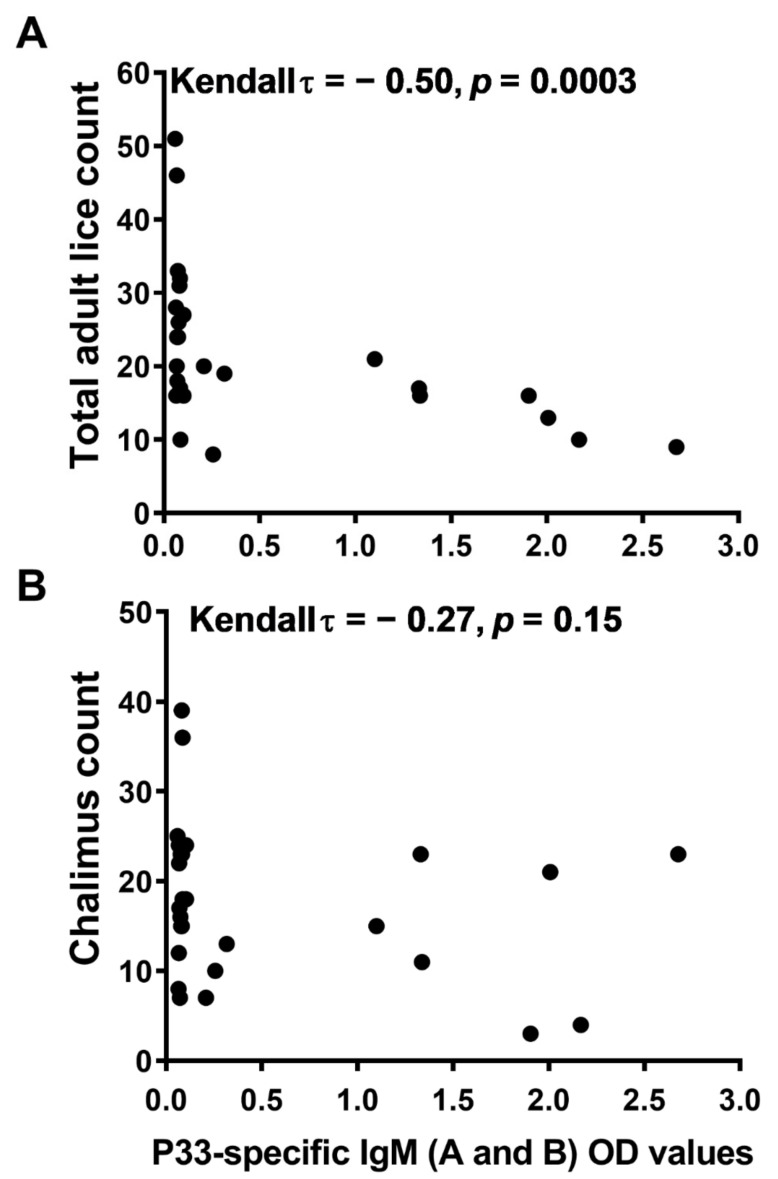
Adult lice count correlates negatively with P33-specific IgM levels in salmon plasma after P33 vaccination. Scatter plot of lice count versus P33-specific IgM (A and B subtypes) levels in blood plasma (OD values by ELISA; *x*-axis), showing the correlation between P33-specific IgM (A and B subtypes) OD values in the plasma of mock-vaccinated (injected with phosphate-buffered saline [PBS]) and P33-vaccinated (high dose [HD]) Atlantic salmon at 18 weeks post-vaccination [wpv], and the lice count on the same fish. (**A**) Scatter plot of total adult lice count (18 wpv); (**B**) Scatter plot of chalimus count (16 wpv). Kendall’s rank correlation coefficients (τ) and *p* values are shown on the plots.

Assessing data from the vaccination trial using Kendall’s rank correlation test showed that the plasma levels of P33-specific IgM exhibited a moderate, negative correlation with the total adult lice counts (Kendall τ = −0.50, *p* = 0.0003; Figure 9A). A similar correlation could not be observed with chalimus counts (Kendall τ = − 0.27, *p* = 0.15; Figure 9B).

## 4. Discussion

The present study reports that vaccination with the recombinant salmon louse protein-potassium chloride and amino acid transporter (P33) resulted in a significant reduction in lice counts during the subsequent experimental challenge of Atlantic salmon (Figure 2A,B). The vaccine with the highest P33 content (HD) reduced the number of chalimus by 41.3% and the number of adult lice by 35.7% as compared to a mock vaccination using PBS with an RPP against adult females estimated at 28.5% in the HD group. The reduction in lice female counts in the HD group in the current study is comparable to that previously reported by Contreras et al. [24], showing a 35% reduction in adult females after P33 vaccination. The protection against sea lice seemed to be correlated to P33 dose since there were significantly lower adult lice numbers in the fish group with high vaccination dose when compared to those with a low dose.

After vaccination, P33-specific IgM antibodies were detected in the plasma of P33-vaccinated salmon, with higher levels in the HD group than in the LD group (Figure 3A), suggesting a dose-response effect of the P33 vaccination. The P33-specific IgM analysis showed a non-significant lower titer on lice-infested fish at 18 wpv (28 days post-infestation[dpi]), as compared to lice-free fish at 14 wpv, which is probably a temporary response of immunoglobulin to lice infestation. In a previous lice challenge trial in Atlantic salmon, Skugor et al. [32] also showed hyporesponsiveness in several immunoglobulin-like genes at 22 dpi as part of a general wave of immunosuppression in response to lice infestation. The immunoglobulins suppression was, however, short and was reverted in 11 days. The production of P33-specific Ab is also supported by the P33-specific labeling identified in situ in the spleen of P33-vaccinated fish in the In.IHC (Figure 4). Although we did not measure P33-specific IgM in skin mucus in the trial presented in this study, we had demonstrated in another P33 vaccination trial (data not shown) that P33-vaccinated salmon can have P33-specific IgM in their skin mucus. This unpublished observation is similar to the results of previous studies showing the ability of salmonids to produce antigen-specific antibodies in skin mucus in response to intraperitoneal vaccination [33,34].

The presence of P33-specific IgM in plasma was not reflected by increased levels of *sIgM* mRNA (qPCR) in the anterior kidney and spleen of P33-vaccinated fish. Indeed, transcript levels of *sIgM*, as well as of the secretory IgT genes, *sIgT-A* and *sIgT-B*, were significantly lower in all analyzed tissues in vaccinated fish as compared to PBS mock controls (Figure 5). This may suggest a downregulation in the expression of the sIg genes in B cells residing in the tissues or, perhaps more likely, a migration of sIg+ cells out of these tissues in response to vaccination. It has been shown that lymphocytes migrate to the site of vaccine injection in Atlantic salmon from 2 weeks after vaccination [35]. In line with this, the number of IgM^+^ cells were lower in the spleen and anterior kidney of P33 vaccinated fish, as judged by scoring of IHC sections(Figure 8A).

The finding that HD group showed higher average of *mIgM* expression in anterior kidney (likely due to a myriad production of naive B cell [rich in surface IgM] to maintain homeostasis after vaccination [36]) at 14 wpv, as compared to pbs group, was not reflected by an increase in IgM+ cells in IHC. This is probably because the F1-18 mAb used to identify IgM in IHC cannot distinguish between the membrane and secreted IgM and that part of the signal identified in some of the IgM+ cells were, perhaps, originated from the secreted IgM molecule in the cells before it was exocytosed. The F1-18 Ab was raised against purified IgM from rainbow trout plasma and the specific ligand it recognizes on the IgM molecule is not yet identified. The Ab has been, nevertheless, found to react with carbohydrate moieties on rainbow trout IgM in WB and has been confirmed to bind to both IgM-A and -B isotypes in Atlantic salmon [29].

In comparison to the change in the mRNA levels of *sIgT-A* and *-B* in the three analyzed tissues (anterior kidney, spleen and skin), the third IgT sub-isotype, *IgT-C*, had significant downregulation of transcripts only in the skin (Figure 6), possibly suggesting a different regulation pattern for this antibody. A previous study has also shown that independent mechanisms might be regulating the involvement of the three trout immunoglobulins (IgM, IgT and IgD) in response to Proliferative kidney disease [37]. Taken together the significant response of *IgT-C* transcripts in skin and the potential secretory nature of IgT-C protein (as predicted based on its truncated heavy chain), a pivotal mucosal immune role of this sub-isotype in salmon after vaccination can be suggested.

The correlation analysis between P33-specific IgM levels in salmon plasma and lice infestation rate of adult and immature lice stages was more significant in the case of adult lice (Figure 9), likely because the immature stages are less dependent on salmon blood for feeding [38]. This pattern of correlation was also seen in other vaccination trials with bigger sample sizes (data not shown), thus supporting the suggestion that protection against the adult lice is mediated by specific Ab. This suggestion is supported also by observations made in two other studies: (i) in a vaccination study against *L. salmonis* in Atlantic salmon, protection against lice using a water-in-oil formulation of the my32-Ls antigen was found to be correlated with antibody titer against my32-Ls [18]; also, (ii) in mammals, protection efficiency against cattle tick using a vaccine formula of tick Bm86 antigen was positively correlated with the Bm86-specific antibody levels in cattle blood [39]. On the other hand, the protection against chalimus is probably mediated by specific Ab in skin mucus of P33-vaccinated fish. Although with a different parasite and in a different fish species, it has been shown that specific antibodies in the mucus of skin [40] and olfactory organs [41] of rainbow trout could protect against the white spot disease caused by the *Ich* parasite (*Ichthyophthirius multifiliis*).

It is yet to be studied how well salmon immunoglobulins can maintain their integrity in the lice gut and whether they can be transported across lice cellular membranes or not [42]. Assuming that antibodies from vaccinated salmon blood can reasonably perform in lice gut, it can be speculated that P33-specific antibodies mediate protection against salmon lice by directly impairing P33 function in situ in the salmon lice gut. The *in-silico* analysis of the amino acid sequence of P33 has revealed its relation to a group of proteins with activities in potassium chloride and amino acid transportation [24]. We, therefore, suggest that the observed protection against salmon lice after P33 vaccination is linked to a disturbance in cellular hypotonic salinity response and transmembrane transportation activity. However, as the retro migration of functional salmon immune components (those engulfed by lice upon feeding) from lice gut to fish has not yet been proved, it is not expected that p33-vaccinated fish can mount a secondary wave of strong immune response upon consequent exposures to sea lice. Efficient protection against sea lice will, therefore, necessitate a constant high titer of post-vaccination P33-specific antibodies in fish blood.

## 5. Conclusions

Here, we demonstrated a dose-response protective effect of P33 vaccine in Atlantic salmon against salmon lice. Our results provided evidence of protection efficiency against both immature and adult lice stages. Intraperitoneal injection with P33 vaccine provoked the salmon immune system and resulted in a specific response against the P33 antigen. The levels of P33-specific antibody produced in fish blood correlated negatively with adult lice counts on fish skin. The protection result against salmon lice is, so far, laboratory-based, and vaccination trials under field conditions typical of salmon farms should be performed before the vaccine can be validated for commercial use.

## Figures and Tables

**Table 1:** Sequence of oligonucleotide primers used in real-time PCR.

Gene Name	Primers Sequences (5′–3′)	Accession Number	Effeciency (%)
Secretory immunoglobulin M (*sIgM*)	F:CTACAAGAGGGAGACCGGAG R:AGGGTCACCGTATTATCACTAGTT	XM_014203125	100.36
Membrane immunoglobulin M (*mIgM*)	F:CCTACAAGAGGGAGACCGA R:GATGAAGGTGAAGGCTGTTTT	Y12457	81.84
Immunoglobulin T isoform C (*IgT-C*)	F:GCTAAGAGTGTCTGGGAAATGA R:TGGAGGGTTTGAGATTGGTC	HQ379938.1	84.9
Immunoglobulin D (IgD)	F:TGAACATCGCTGCTTCAAC R:CCAGCACAGCACTGTCTCC	AF141606.1	97.09
Secretory immunoglobulin T isoform A (*sIgT-A*)	F:CCAAGGATAAGTGGGAGAGAA R:TCACTTGTCTTCACATGAGTTACC	GQ907003	80.2
Membrane immunoglobulin T isoform A (*mIgT-A*)	F:CCAAGGATAAGTGGGAGAGAA R:AGGATGTTCGCCATGGACT	GQ907003	103.41
Secretory immunoglobulin T isoform B (*sIgT-B*)	F:GAATGTTTGGGACACGGAAG R:TCACATATCTTGACATGAGTTACCC	GQ907004.1	89.89
Membrane immunoglobulin T isoform B (*mIgT-B*)	F:GAATGTTTGGGACACGGAAG R:GCTCAGTCAGTGGGATGTTCT	GQ907004.1	92.3
Elongation factor-1alpha *(EF-1 α*)	F:TGCCCCTCCAGGATGTCTAC R:CACGGCCCACAGGTACTG	BG933897	106.98

**Table 2:** Scoring scale of secreted IgM and IgM^+^ cells in spleen and anterior kidney after P33 vaccination.

Tissue	Scoring Scale	Score Description
**Spleen and anterior kidney**	**Abundance of IgM+ cells/microscopic field (10× magnification objective lens)**	
	0	No positive cells
	1	≤10 cells
	2	30–50 cells
	3	60–80 cells
	4	>90 cells
		**IgM labeling intensity**
	0	No labeling
	1	Mild labeling
	2	Moderate labeling
	3	Strong labeling
	4	Heavy labeling

## Supplementary Materials

The following are available online at https://www.mdpi.com/article/10.3390/vaccines10010016/s1, Figure S1: In.IHC negative control spleen sections showed no specific signal for P33-specific antibodies. Figure S2: IHC negative control spleen and anterior kidney showed no specific signal for IgM. Table S1 shows the analyses performed on fish blood and tissue samples and the groups and the number of fish used per each analysis.
