# Supplementary material for: Protective Immunization of Atlantic Salmon (Salmo salar L.) against Salmon Lice (Lepeophtheirus salmonis) Infestation
Source: Vaccines (Basel). 2021 Dec 23;10(1):16. doi: 10.3390/vaccines10010016 (PMC8780844; doi:10.3390/vaccines10010016)
Supplement: Supplementary file 1 [file vaccines-10-00016-s001.zip › vaccines-1423145-supplementary.pdf]

Supplementary Table S1. Shows the analyses performed on fish blood and tissue samples and the groups and the number of fish used per each analysis.

	Samples collected and analyzed at different time points (WPV)											
	7				14				18			
**Analyses**	PL	AK	Sp	Sk	PL	AK	Sp	Sk	PL	AK	Sp	Sk
**Specific P33 Ab in plasma (n≥10/group)**	●♦■				●♦■				●♦■			
**Inverted IHC (n≥10/group)**							●■					
**Immune gene expression (n≥7/group)**		●■	●■	●■		●■	●■	●■		●■	●■	●■
**IHC analysis (n≥8/group)**						●■	●■					
**Correlation between P33 specific IgM and sea lice count (n≥12/group)**									●■			

WPV: weeks post-vaccination**, PL:** plasma**, AK:** Anterior kidney**, Sp:** spleen, **SK:** skin**,** PBS group (●), P33 LD group (♦) and P33 HD group (■).

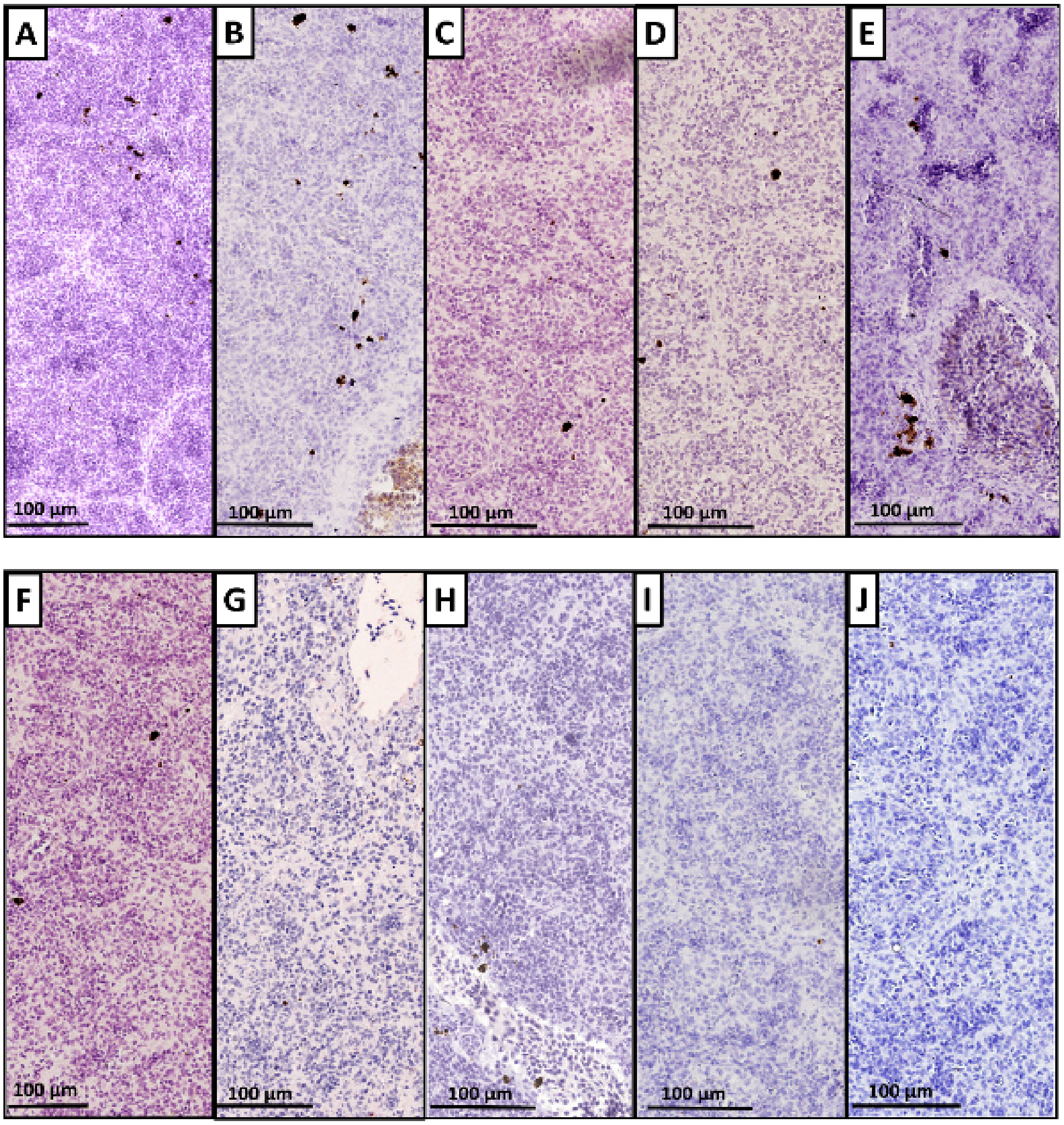

Supplementary Figure S1. In.IHC negative control spleen sections showed no specific signal for P33-specific antibodies.. Images of spleen collected from fish in P33 HD (A- E) and pbs (F - J) group at 14 wpv, represent sections incubated with HIS-tagged recombinant P30 protein (A and F), incubated with HIS-tagged recombinant P12 protein (B and G), omitting only P33 bait (C and H), omitting only secondary antibody (D and I), or omitting only HRP labeled anti-rabbit antibody (E and J).

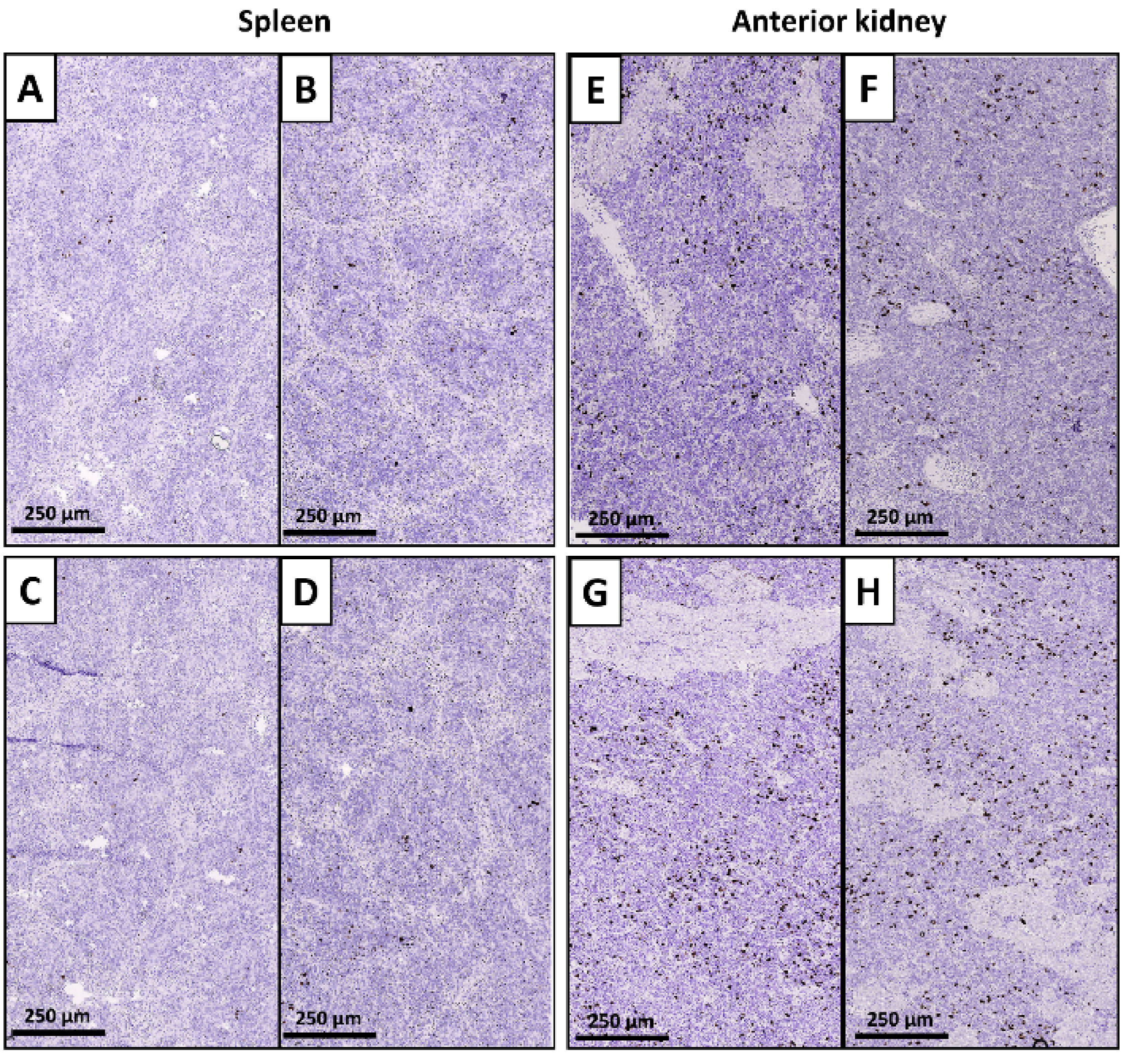

Supplementary Figure S2. IHC negative control spleen and anterior kidney showed no specific signal for IgM. Images of spleen (A – D) and anterior kidney (E – H) obtained from the pbs (A, B, E, and F) and P33 HD (C, D, G, and H) group at 14 wpv represent sections omitting only anti-IgM antibody (A, C, E, and G) or omitting only HRP labeled anti-mouse antibody (B, D, F, and H).
